# The Effect of Gastrointestinal Nematode Infection Level on Grazing Distance from Dung

**DOI:** 10.1371/journal.pone.0126340

**Published:** 2015-06-03

**Authors:** Hizumi Lua Sarti Seó, Luiz Carlos Pinheiro Machado Filho, Luciana Aparecida Honorato, Bruna Fernanda da Silva, Alessandro Fernando Talamini do Amarante, Patrizia Ana Bricarello

**Affiliations:** 1 Laboratório de Etologia Aplicada e Bem-Estar Animal, Departamento de Zootecnia e Desenvolvimento Rural, Universidade Federal de Santa Catarina, Florianópolis, Brazil; 2 Universidade do Planalto Catarinense, Lages, Brazil; 3 Departamento de Parasitologia, Instituto de Biociências, UNESP—Universidade Estadual Paulista, Botucatu, Brazil; 4 Laboratório de Parasitologia Animal, Departamento de Zootecnia e Desenvolvimento Rural, Universidade Federal de Santa Catarina, Florianópolis, Brazil; Linneaus University, SWEDEN

## Abstract

Avoiding grazing near feces is an efficient strategy to prevent parasitic infection and contamination; therefore, in the evolution of herbivorous species, this behavior may have developed as a mechanism to protect the host against infection by gastrointestinal nematodes. The aim of this study was to assess whether grazing distance from dung is related to the level of parasitic infection in cattle. Based on Fecal Egg Count (FEC) means, 18 castrated male steers, aged 18 months, were divided into three groups: High (FEC ≥ 315); Medium (FEC = 130–160); and Low (FEC = 40–70). To analyze the response to a new natural infection by gastrointestinal nematodes and to standardize infection levels, all animals received anthelmintic treatment at twenty days prior to field observation. Three observers simultaneously collected data on grazing behavior for 2.5 hours/week for 12 weeks. Observers recorded the distance when grazing occurred at less than one meter from dung. Every two weeks, fecal samples were collected for FEC, as well as serum samples to measure immunoglobulin G (IgG) levels against larvae and adult antigens of the parasitic species *Haemonchus placei*. All groups grazed farther from the dung on days of greater insolation (r = 0.62; P = 0.03). Animals with high levels of parasitism grazed farther from the dung (P < 0.05) but had lower levels (P < 0.0001) of IgG serum levels compared to those with medium and low levels of infection. FEC values varied over the experiment, remaining below 200 for the low and medium group and reaching 1000 (P < 0.01) for the animals with the highest rates of parasitism. Our results indicate that cattle showing high levels of parasitism are more likely to avoid contaminated areas than animals with lower infection levels, and the immune system seems to be involved in such behavior.

## Introduction

The production potential of grazing cattle can be compromised by factors that are frequently related to animal health. Parasite control in ruminants has several benefits in relation to productivity, including weight gain, improved feed conversion, increased milk production, better reproductive performance, greater carcass quality, improved immunological status, and reduced morbidity and mortality [1,2].

Losses caused by worm infections in countries like the United States have accounted for around $330 million USD/year. According to the National Union of the Industry of Animal Health Products, 56% of the sector revenue in Brazil in 2011 originated from products for ruminants, of which 24% were antiparasitics [3]. However, farmers have used these drugs intensively and without a strategic focus [4], leading to rapid development of anthelmintic resistance [5–7] and residues in meat products. As a result, the Brazilian Ministry of Agriculture, Livestock and Supply prohibited the use of avermectins [8]. Therefore, it is essential to study alternative strategies to control worm infection. In some parts of the world, such as New Zealand, genetic selection of resistant animals has been evaluated and is currently in use [9].

Some phenotypic markers such as the number of eggs per gram of feces (FEC), packed cell volume, number of circulating eosinophil, and antibody levels have been associated with parasite resistance and can be used as parameters in selection programs [10,11]. Thus, gaining a better understanding of immune mechanisms may contribute to new perspectives on increasing resistance to gastrointestinal nematodes in breeding programs.

Considering that gastrointestinal parasite infections are known to reduce the chances of survival in natural selection, the ability of the host to avoid ingestion of these parasites is critical for its survival; thus, if this behavior is a genetically determined trait, it will be passed on to offspring [12]. In fact, parasitism represents a significant challenge for the host in relation to survival and reproduction and is expected to have exerted a significant evolutionary pressure on the host's behavior to combat its effects [13]. Sheep, for example, that are more resistant to nematodes have been selected for over the past several decades and this has resulted in animals with different immunological and behavioral characteristics [14]. Fecal avoidance in herbivores is believed to limit fecal-oral transmission of pathogens, consequently lowering the risk of infection [15]. Thus, behavioral adaptations may be part of the host's means of controlling parasites.

Ruminants cannot detect nematode infective larvae on the grass; one strategy to reduce ingestion of larvae may be to avoid foraging near feces. Animals can detect the presence of feces through smell and visual clues, such as the color and height of pasture [14]. In general, herbivores avoid foraging in areas contaminated with dung, especially if the dung is of their own species [16]. Horses, for example, avoid grazing patches of tall grass, which are generally of low quality, selecting short patches located at least 1 m from feces [17]. Cattle in rotational grazing systems avoid grazing closer than 0.5 m from feces up to 63 days after defecation [18]. Other studies have shown bovine species avoiding the consumption of food smelling of feces, as the odor is perceived by the animal’s sense of smell [19,20].

There are few studies assessing animal behavior as a parameter for genetic selection related to parasite resistance, particularly for cattle. In addition, the specific selection of beef cattle resistant to endoparasites has not been conducted, but resistance and other individual traits could be related to animal behavior. Thus, the aim of this study was to assess whether the distance of foraging behavior from dung is related to the level of natural gastrointestinal parasite resistance in cattle.

## Material and Methods

### Animals and experimental design

The experiment was conducted between February and May 2012 at the Federal University of Santa Catarina (UFSC) "Ressacada" Experimental Farm, Florianopolis, SC, Brazil. Eighteen castrated European crossbred steers were selected for the experiment out of 30 animals, all aged 18 months; the selection was based on mean nematode Fecal Egg Counts (FEC), which were determined for each animal at 15-day intervals over the 145 days before the experimental phase. Steers had natural nematode infection with the genera *Haemonchus*, *Oesophagostomum*, *Trichostrongylus* and *Cooperia*. The six most infected animals, the six least infected animals, and six animals showing medium levels of infection were allocated to three groups:

- Low: the six animals with the lowest levels of infection, mean FEC between 40 and 70;
- Medium: six animals with medium levels of infection, mean FEC between 130 and 160;
- High: the six animals with the highest levels of infection, mean FEC above 315.

To analyze an initial response to natural gastrointestinal nematode infection and to standardize the infection level, all animals received anthelmintic treatment with albendazole (10 mg/kg, Aldazol 10 CO; Vallée S.A., Sao Paulo, Brazil) 20 days prior to observation, reducing the FEC to zero. The methods used in this study were approved by the UFSC Ethics Committee on Animal Use, protocol number PP00673, and an accredited veterinarian collected blood and fecal samples.

Although we selected only 18 animals for the study, all 30 animals remained together throughout the experiment. We weighed each animal included in the study at the beginning and end of the experimental phase. The steers were kept on pasture throughout the duration of the study and had continual access to water, mineral mixture, and shade. The total area of the multispecies permanent pasture was 26 ha, where Limpo grass (*Hemarthria altissima)* and Bluestem grass (other Gramineae of the genus *Andropogon* spp.) are predominant.

The experimental area contained six paddocks of one hectare each, managed with rotational grazing. Immediately before the start of the experiment, all paddocks were mowed to standardize the pasture. Data collection occurred at 7- or 14-day intervals to ensure 25 days of rest for each paddock. The animals remained in each paddock for four days; to ensure a reduction in the sward height for better visualization of the fecal patches, data were collected on the fourth day in each paddock. During data collection, the sward was regular and approximately 10 cm high. Any mosaic formed during the experiment was related to patches around feces avoided by the cattle.

We obtained local meteorological data (insolation, temperature, rainfall, and relative humidity) throughout the experimental phase from meteorological station 83897 of the National Institute of Meteorology (INMET) (latitude -27.58°, longitude -48.56°, altitude 1.84 m), located in the metropolitan region of Florianopolis, SC, Brazil.

### Measurements

#### Behavioral observation and pasture larva collection

Observations of all 18 animals were recorded every Monday for 12 weeks, from 8:00 to 10:30 am. Three evaluators observed and recorded the grazing behavior of animals based on the distance from dung. Six animals with varying levels of infection were randomly allocated to each observer to prevent observer effect. Each evaluator observed each animal separately, as a focal animal, for five minutes. If during the first three minutes grazing occurred at less than 1 meter from dung, in the two remaining minutes the exact distance from the fecal patches of the closest bite was recorded in centimeters using a tape measure. Pasture was collected from the exact grazed site to estimate the number of ingested infective larvae (L3). The one-meter distance is based on our previous research, in which the minimum grazing distance from the dung border was found to be 0.4 m [18]. Therefore, we extended the observation radius to 1m for the ease of using an integer and to define an area that likely represents a grazing patch or the minimum range where selective grazing occurs. If the minimum distance is 0.40 m, grazing farther than one meter is unlikely related to the avoidance of fecal matter.

To collect pasture at the grazing site closest to the dung, a 20cm-radius hoop was placed exactly where the animal grazed and all pasture within the hoop area was cut to 3 cm above the soil. The material was stored in plastic bags, properly identified with the distance from dung, observed animal, paddock, and sampling date. Grass samples were weighed and L3 recovery from the pasture was performed by sedimentation in buckets, as described by Rocha et al. [21], and expressed as number of L3 per kilogram of pasture dry matter (L3/kg). The larvae were fixed, stained with Lugol’s iodine, and identified based on descriptions provided in Keith [22].

All observers were trained at the beginning of the experiment to ensure inter-observer reliability [23,24]. During training sessions, the animals were acclimatized to the presence of observers inside the paddock to avoid interference during observation. Animals were identified by ear tags, coat color, and numbers written on their sides using livestock markers (Raidex, Dettingen/Erms Germany).

#### Fecal examination

After observation and sampling of the consumed pasture, the study animals were directed to the containment chute for individual fecal collection directly from the rectum for FEC [25] and fecal cultures [26]. The collected infective larvae were then identified as described above [22].

#### Weight gain

Body weight was estimated at the beginning and end of the experiment by the same individual using a girth tape placed around the animal’s chest, behind the elbow joints. All animals had their weights estimated using the scale on the tape and average weight gain was calculated for each group.

#### Enzyme-linked immunosorbent assay (ELISA)

Blood samples were collected without anticoagulant using coccygeal artery puncture. Serum samples were stored at -20°C for subsequent immunoglobulin G (IgG) analysis. Serum IgG levels against *Haemonchus placei* L3 and adult antigens were measured in samples collected at 8, 22, 36, 50, 64, 78, 102 and 116 days after the beginning of the study. Antigens were obtained following the procedure outlined by Bricarello et al. [27].

Total IgG levels in the sera were measured by ELISA (enzyme-linked immunosorbent assay), as described by Cardoso et al. [28]. Briefly, parasite-specific serum IgG polystyrene micro-titer plates (Nunc, Rochester, NY, USA) were coated with *H*. *placei* antigens and the plates were incubated overnight at 4°C. All subsequent incubations were carried out for 1 h at 37°C. Between each step, plates were washed three times. After coating, blocking was carried out and serum samples were diluted and applied in duplicate. We then incubated the plates with peroxidase-conjugated sheep anti-bovine IgG diluted at 1:10,000 (A10-118P; Bethyl Laboratories, Montgomery, TX, USA). Finally, 1,2-phenylenediamine dihydrochloride (OPD) substrate solution (Dako, Glostrup, Denmark) was added to each well and the enzymatic reaction was allowed to occur. Plates were read at 492 nm using an automated ELISA reader (Biotrak II; Amersham Biosciences, Little Chalfont, UK). IgG results were expressed as a percentage of optical density of the positive-reference serum [29].

### Statistical analyses

To compare FEC before and after de-worming for each animal, we used a simple paired-t test. For all other analyses, we used the following methods: mixed model with fixed effects of infection level and interaction between infection level and data collection date; effect of date as a repeated measure and random effect of animal, nested under infection level; error for the effect of infection level and residue using MIXED procedure of SAS, version 9.0 [30]. Of all assessed structures of error, compound symmetry structure (CS) was the most appropriate, according to the Bayesian information criterion (BIC). The command LSMEANS was used to separate means. A correlation test among evaluators was performed to standardize observations. We transformed FEC and IgG results using log10 (x+1) to stabilize the variance before statistical analysis. For all analyses, we set significance at P ≤ 0.05 and calculated Pearson’s correlation with the transformed data. All results are expressed as arithmetic means (± standard error) of back-transformed data.

## Results

### Foraging behavior

**Fig 1:**
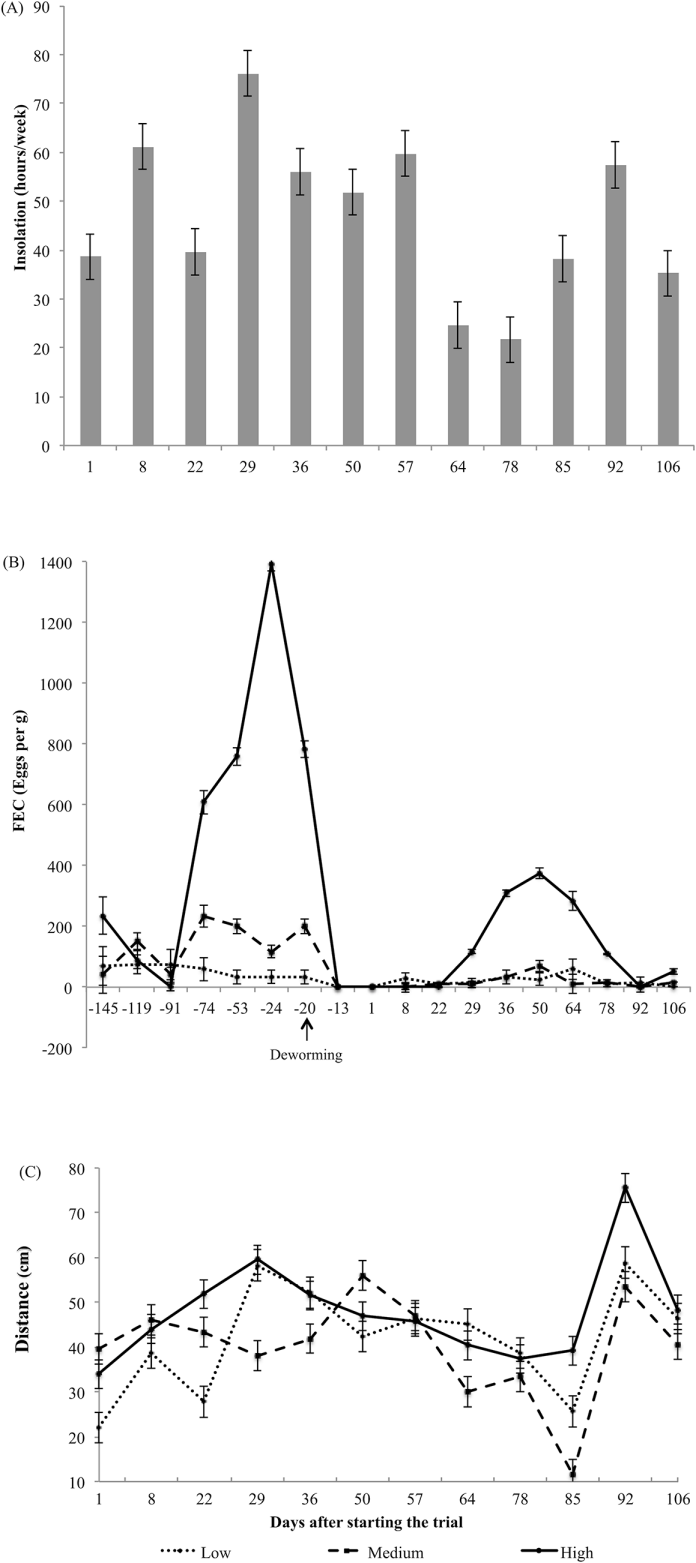
Variables in days after starting the trial. (A) Insolation (hours/week) during the experimental period (Source: adapted from INMET, 2012). We found a correlation between grazing distance from the dung and insolation (P = 0.03). (B) Fecal Egg Counts (FEC) for the three cattle groups. Twenty days after administration of anthelmintic (indicated by an arrow), we found greater values for the High group than Low and Medium (P < 0.01). Data are absolute FEC (±) SE. (C) Mean distance between dung and the grazing site by group. Bar represents arithmetic means (±) SE. Results show a difference between groups (P = 0.05) and days (P < 0.0001).

The mean grazing distance from dung was greater for the High group (mean of 50 cm) than for the Medium (mean of 41 cm; P = 0.02) and Low (mean of 42 cm; P = 0.05; Fig 1C) groups.

Our results show that animal behavior was influenced by climatic conditions (Fig 1A). Even though the animals with the highest parasite levels grazed farther from the dung, all three groups (Low, Medium and High) showed similar behavioral patterns in relation to insolation; all groups avoided grazing close to dung during weeks of greater insolation. We found a positive correlation between the mean distance from dung and insolation (r = 0.62; P = 0.03).

### Larva collection from pasture

We detected no difference among groups for L3 quantities in the collected pasture samples (mean of 99, 139, and 45 L3/kg/MS for Low, Medium, and High, respectively; P > 0.05). The predominant genera on the pasture were *Haemonchus* (39%) and *Oesophagostomum* (40%), followed by *Trichostrongylus* (11%) and *Ostertagia* (10%). *Cooperia* was not found in the pasture samples.

### Fecal examination

The average FEC before the experiment was significantly higher (p<0.001) than after for all animals; however, we found a strong correlation (r = 0.71; P < 0.001) between pre- and post-albendazole FEC values among animals. While FEC values varied over the experiment, the Low and Medium groups retained low FEC levels, ranging from 0 to 200 (mean FEC of 17). The animals in the High group presented the highest FEC means, peaking at day 50 (400 FEC) and progressively declining to values close to zero at the end of the study (P < 0.01) (Fig 1B).

Throughout most of the study, *Haemonchus* (62%) was the predominant genus found in composite cultures, followed by *Oesophagostomum* (18%), *Trichostrongylus* (17%), and *Cooperia* (3%). *Ostertagia* was not identified in the coprocultures.

### Weight gain

The infection level did not influence weight gain of the studied cattle. The initial and the final average weight was 251 and 303 kg, respectively. The Low, Medium, and High groups had average weight gain of 51.8, 60.8, and 42.7 kg, respectively. We only detected a tendency (P = 0.09) towards greater weight gain for moderately infected animals, compared to highly infected ones. There was a strong correlation between the initial and final weight.

### Enzyme-linked immunosorbent assay (ELISA)

**Fig 2:**
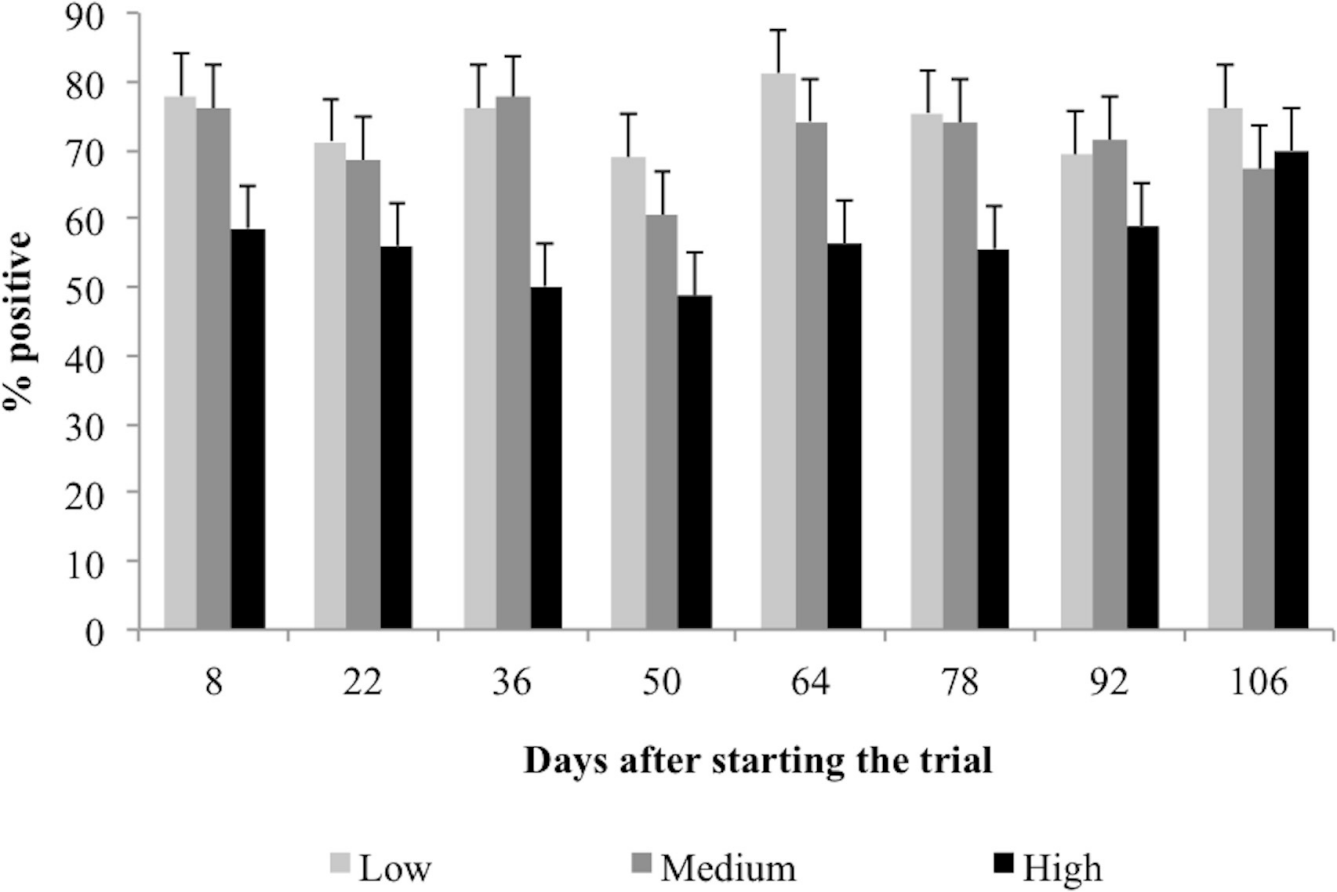
Mean percentage of *H*. *placei*-specific serum IgG levels against L3 worm crude antigen for groups Low, Medium, and High showing Fecal Egg Counts (FEC). Data are the mean of duplicates (±) SE given as a percentage of optical density of the positive reference serum. There was a difference between groups (P = 0.03) and days (P = 0.002) for IgG against L3 but no significant (P < 0.05) interaction between groups and days.

Except for the sample taken in the final week, the total serum IgG levels against *H*. *placei* larvae antigens was significantly lower for the susceptible group (High), compared to the resistant groups (Low and Medium); means were 56%, 71%, and 74% of optical density for the positive-reference serum, respectively (P < 0.0001; Fig 2). No significant difference (P < 0.05) was found between groups for adult-specific IgG. Our results demonstrate an inverse relationship between serum IgG levels against *H*. *placei* larvae antigens and FEC; animals showing lower IgG production had a higher level of gastrointestinal nematode infection (r = -0.28; P < 0.01).

## Discussion

The aim of this study was to assess whether the behavior of grazing distance from dung is related to the level of gastrointestinal parasite infection in cattle. Due to the administration of anthelmintic, the level of infection decreased significantly in all animals after the start of the experiment, compared to historical infection levels, and all animals built immunity over the course of the study. However, the same animals that showed high FEC levels before parasite treatment retained high infection levels later on in the study. The infection level of the Medium group decreased after deworming, approaching that of the Low group. We hypothesize that the Low group had acquired some level of resistance after a period of exposure [31]. Animals with high levels of parasite infection grazed farther from dung compared to animals with lower levels of infection, which is consistent with results found in studies involving sheep [32–34].

A possible explanation for this behavior is based on the hypothesis that parasitized individuals have the ability to determine their parasitic status and change their behavior to prevent further infection levels because of their inefficient immune response [35]. The physiological mechanism involved is still unknown but it could be complementary to other behavioral strategies of hosts to avoid infection, such as searching for food with anthelmintic effects [36].

Szyszka and Kyriazakis [37] note that only animals with high levels of L3 infection show behavioral differences and only at a certain period after infection, until larvae matures. Lower levels of infection do not cause behavioral changes. Therefore, even though abomasal damage was not observed, it is possible that the decision to graze farther from the feces only occurs when the host's abomasal damage is above a certain threshold related to pain or discomfort. This could explain the lack of observed behavioral difference between animals in the Low and Medium groups.

Hutchings et al. [14] demonstrate that in comparison to susceptible animals genetically resistant sheep graze farther from the clumps, which potentially house a larger number of larvae. The authors suggest that genes that determine the foraging behavior may be co-selected in the process of genetic selection for immunity. However, beyond the immunological phenomena, rejection to parasites may involve other processes, such as mechanical or enzymatic damage of parasites inflicted by the host as well as nutritional state, which complicates the host's behavioral response.

In addition to the relationship between behavior and parasitism, we observed the animals grazing farther from the dung during greater levels of insolation. This finding suggest that, as with other behaviors [38], their grazing strategy when close to dung changes according to environmental conditions [32]. This behavior is related to the heat caused by insolation that makes the repellent compounds in the dung more potent. In fact, smell is a major sense involved in grazing behavior [39] and cattle identify and avoid volatile chemicals from feces up to 35 days after excretion [19], maintaining a distance of at least 0.5 m from the fecal patches for 63 days [18].

Dohi et al. [19,40] determined that the volatile neutral fraction is a deterrent for animals and assumed that these odor compounds in the feces are influenced by weather conditions on the pasture. In fact, similar to the transference of energy to molecules leading to greater water evaporation during insolation, we suggest that the same process occurs in the dung making the odor more pungent and this is sensed by the animals, keeping them away from dung patches.

The difference in results for mean grazing distance from dung for animals in the Low and High groups is apparently small. However, this difference becomes more significant when we consider that 90% of larvae are found within 10 cm of the feces and this value logarithmically increases moving closer to the feces [41]. For directly transmitted parasites that rapidly develop on the pasture, fecal avoidance can decrease the risk of infection [42], including the risk of *Haemonchus* infection in tropical areas.

Regarding weight gain, the absence of difference among groups is probably due to the use of anthelmintic treatment and the duration of the study which was insufficient to assess the negative effects of parasitism on the performance of the most highly parasitized animals. Furthermore, the relationship between resistance to parasitism and body weight is still controversial. Some studies have indicated that resistance in sheep has a negative genetic correlation with productive characteristics such as growth [43], while others have found a positive genetic correlation between resistance to parasitism and body weight [44].

The Low and Medium groups retained low FEC and we did not find behavioral differences between the two groups. Furthermore, the groups showed similar immunoglobulin (IgG) levels. Therefore, these two groups formed a homogeneous group with low levels of parasitism, despite the fact that the animals were initially classified into separate groups based on levels of parasitism. However, the acquired immune response is a dynamic process. Animals showing high infection levels at the beginning and low IgG levels against L3 throughout the study remained the same, therefore justifying the phenotypic plasticity in the grazing behavior.

The High group grazed farther from the dung and had higher FEC values than the other two groups. A possible explanation is that the low immunity of the cattle, as shown by the lower serum IgG levels, may lead to increased larvae in the gastrointestinal tract of these animals, which in turn changes their behavior to avoid contaminated sites. Over a several-week study, Zaros et al. [45] noted higher L3-specific IgG1 levels for resistant Nelore cattle in comparison to susceptible cattle. They also found higher adult-specific IgG1 levels at 84 days after the start of the trial with young Nelore bulls naturally infected by *Haemonchus*. Resistant cattle also produced higher levels of *Cooperia*-specific serum IgG1 than susceptible bulls, which suggests that immunoglobulin plays a role in host protection [27].

Animals with high FEC before deworming presented lower IgG on the days following deworming. This tendency was persistent throughout the entire experiment (Fig 2). Interestingly, the High group was the only group that showed an increase in FEC a few weeks into the study (Fig 1B), which was accompanied by a greater grazing distance from dung. The similar weight gain performance presented across all groups also showed that the animals with high FEC were resilient to infection. They did not present any clinical sign of parasitic gastroenteritis or a reduction in weight gain. A stochastic model formulated by Fox et al. [42] shows that fecal avoidance is an efficient strategy for hosts with lower immune response in the presence of parasites that have rapid development on pasture. On the other hand, the low-level trickle infection received by hosts with no fecal avoidance behavior may be enough to engender an immune response, but not enough to create high levels of parasite establishment.

Although several studies have shown the relationship between genetic resistance and acquired immune response [27,45,46], the present paper describes for the first time the association between foraging behavior and serum IgG levels and further investigation is necessary. Our results suggest an evolutionary advantage of more resistant animals. Although the animals are at a higher risk due to grazing behavior closer to feces, resistant animals have greater pasture abundance due to nutrient leaching to grazing sites, which may increase nutrient ingestion and boost their immune system.

## Conclusion

Our results demonstrate that the animals most affected by parasites graze farther from dung. This is the first study with beef cattle demonstrating the relationship between behavior and immune system; this avoidance behavior is probably an animal strategy aimed at preventing parasitic infection. Insolation also influenced the foraging behavior close to dung patches. Understanding the relationship between resistance and animal behavior may help to select genetically advantageous animals to improve pasture use and reduce the need for antiparasitic drugs.
